# Anthracimycin B, a Potent Antibiotic against Gram-Positive Bacteria Isolated from Cultures of the Deep-Sea Actinomycete *Streptomyces cyaneofuscatus* M-169

**DOI:** 10.3390/md16110406

**Published:** 2018-10-25

**Authors:** Víctor Rodríguez, Jesús Martín, Aida Sarmiento-Vizcaíno, Mercedes de la Cruz, Luis A. García, Gloria Blanco, Fernando Reyes

**Affiliations:** 1 Fundación MEDINA, Centro de Excelencia en Investigación de Medicamentos Innovadores en Andalucía, Avda. del Conocimiento 34, Parque Tecnológico de Ciencias de la Salud, E-18016 Granada, Spain; vrm00014@correo.ugr.es (V.R.); jesus.martin@medinaandalucia.es (J.M.); mercedes.delacruz@medinaandalucia.es (M.d.l.C.); 2 Departamento de Biología Funcional, Área de Microbiología, and Instituto Universitario de Oncología del Principado de Asturias, Universidad de Oviedo, 33006 Oviedo, Spain; UO209983@uniovi.es; 3 Departamento de Ingeniería Química y Tecnología del Medio Ambiente, Área de Ingeniería Química, Universidad de Oviedo, 33006 Oviedo, Spain; luisag@uniovi.es

**Keywords:** anthracimycin, *Streptomyces cyaneofuscatus*, deep-sea actinobacteria, Cantabrian Sea

## Abstract

The potent antimicrobial extract of a culture of the marine derived actinomycete *Streptomyces cyaneofuscatus* M-169 was fractionated by reversed phase flash chromatography and preparative HPLC to yield the new Gram-positive antibiotic, anthracimycin B (**1**), together with its congener, anthracimycin (**2**). The structure of the new compound was established by analysis of its ESI-TOF MS and 1D and 2D NMR spectra, and comparison with data published for anthracimycin and anthracimycin BII-2619 (**3**). Notably, anthracimycin seemed to be the major and almost unique component of the extract detected by HPLC-UV-MS, making our *S. cyanofuscatus* strain an excellent candidate for further biosynthetic studies of this potent antibiotic.

## 1. Introduction

Deep sea actinomycetes are the source of a wide range of new natural products with antibiotic and anti-cancer properties [1]. The macrolide anthracimycin (**2**) was first isolated from cultures of a sediment derived *Streptomyces* sp. CNH365 collected in the Pacific Ocean, off the shore of Santa Barbara (California), and its structure determined by NMR analysis and X-ray crystallography [2]. Since its recent discovery in 2013, anthracimycin became the subject of active research given its extraordinary potent antibiotic activities against *Bacillus anthracis* [3], whose spores have been used as bioterrorism weapons. Anthracimycin is also active against methicillin-resistant *Staphylococcus aureus* [4], and novel activities against human hepatocarcinoma have also been reported [5]. Its biosynthetic pathway, involving a type I polyketide synthase (PKS), was deciphered in different studies [6,7,8]. Very recently an isomer of anthracimycin, designated as anthracimycin BII-2619 (**3**), was identified from a different actinomycete, *Nocardiopsis kusanensis* [9].

**Figure 1:**
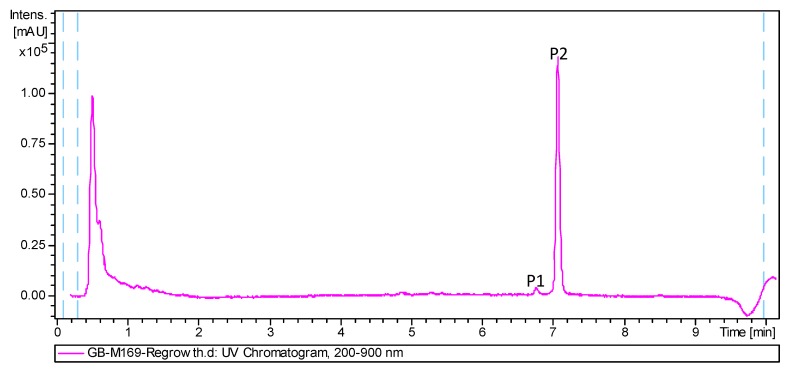
HPLC trace of the ethyl acetate extract of *Streptomyces cyaneofuscatus* M-169.

As part of our program aimed at the characterization of actinomycete strains isolated from deep-sea water and invertebrates (1500–4700 m depth) collected in the Cantabrian Sea [10], we focused our attention in extracts of the strain M-169 of *Streptomyces cyaneofuscatus*, isolated from a gorgonian coral (Order Gorgonacea) collected at 1500 m depth in the Avilés submarine Canyon. New natural products with antibiotic and cytotoxic activities were previously identified from actinomycetes collected in this canyon [11,12,13]. Very recently, the strain M-157 of *S. cyaneofuscatus* was reported by our groups to produce 3-hydroxyquinaldic acid derivatives that might be biosynthetic precursors of chromodepsipeptides with antimicrobial or cytotoxic properties [14]. Herein, the finding of a new compound with potent antimicrobial activities in samples collected from this extreme environment is reported. LC-UV-HRMS dereplication [15] of the antimicrobial ethyl acetate extract of strain M-169 revealed the presence in the chromatographic UV trace (Figure 1) of only two peaks with molecular formulae of C_24_H_30_O_4_ (P1) and C_25_H_32_O_4_ (P2). Dereplication using the latter molecular formula, the UV spectrum, and the Chapman and Hall Dictionary of Natural Products identified anthracimycin (**2**) [2] or its isomer, anthracimycin BII-2619 [9], as the only possible hits. Given the potent and interesting antibiotic activities described for these molecules, including activity against *B. anthracis*, we decided to isolate the two peaks present in the extract to confirm the identity of the major compound (P2) by HRMS and NMR, and to establish the structure of the second component of the extract (P1), whose molecular formula and UV spectrum suggested it to be an analogue of anthracimycin. Fractionation of the extract using reversed phase flash chromatography and preparative HPLC resulted in the isolation of the new natural product, anthracimycin B (**1**), and the confirmation of the identity of the major component of the extract as anthracimycin (**2**). 

## 2. Results

### 2.1. Taxonomy and Phylogenetic Analysis of the Strain M-157

**Figure 2:**
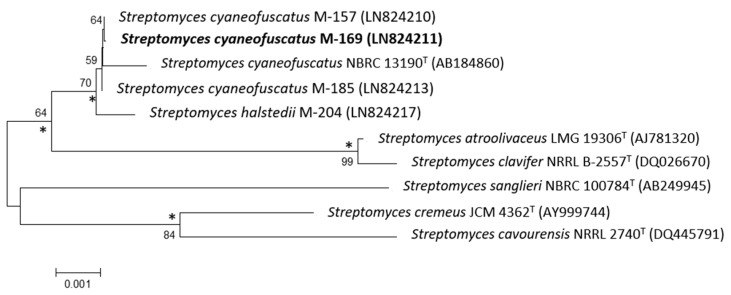
Neighbour-joining phylogenetic tree obtained by distance matrix analysis of 16S rDNA sequences, showing *Streptomyces cyaneofuscatus* M-169 position and most closely related phylogenetic neighbours. Numbers on branch nodes are bootstrap values (1000 resamplings; only values >50% are given). Asterisks indicate that the corresponding nodes were also recovered in the maximum likelihood tree. Bar indicates 0.1% sequence divergence.

The 16S rDNA of strain M-169 was amplified by polymerase chain reaction (PCR) and sequenced [10]. After Basic Logic Alignment Search Tool (BLAST) sequence comparison, strain M-169 showed 100% identity to *S. cyaneofuscatus* (Accession number NR_115383); thus, this strain was designated as *S. cyaneofuscatus* M-169 (EMBL Sequence number LN824211). The phylogenetic tree generated by the neighbour-joining and maximum likelihood method based on the 16S rDNA sequence clearly revealed the evolutionary relationship of strain M-169 with a group of known *Streptomyces* species (Figure 2).

### 2.2. Isolation and Structural Elucidation of Compounds ***1*** and ***2***

**Figure 3:**
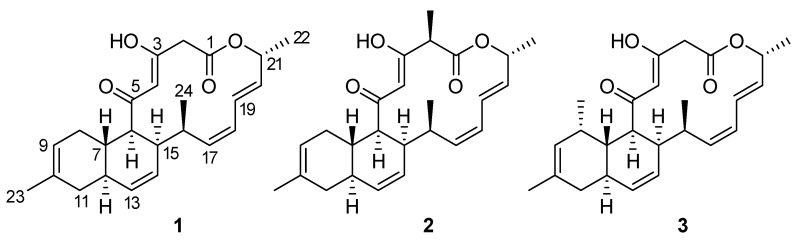
Structures of compounds **1**–**3**.

A culture of *S. cyaneofuscatus* M-169 in solid R5A medium was extracted with acidified ethyl acetate. Fractionation of this extract using reversed phase C18 column chromatography and preparative HPLC yielded the new compound, anthracimycin B (**1**), together with anthramycin (**2**) (Figure 3), whose NMR spectra were identical to those described in the literature [2]. 

**Figure 4:**
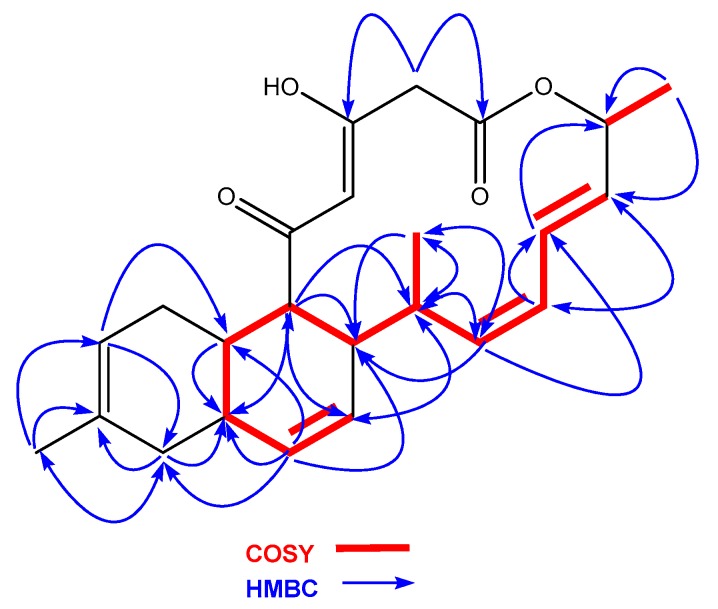
COSY and HMBC correlations observed in the structure of **1**.

Anthracimycin B (**1**) was isolated as a white amorphous solid, whose molecular formula, C_24_H_30_O_4_, was deduced from the presence of a protonated ion at *m/z* 383.2220 in its ESI-TOF mass spectrum (calc. for C_24_H_31_O_4_^+^, 383.2217). This molecular formula together with UV absorption maxima at 236 and 288 nm were compatible with an anthramycin-like structure for the molecule. Chemical shifts measured in its ^1^H and ^13^C NMR spectra (Table 1) corroborated this structural similarity. Comparing the 1D and 2D NMR spectra with those published for anthracimycin [2], the major differences were found in the region around carbons, C-1 to C-3, where the methyl group attached to C-2 in **2** was absent in the structure of **1**. Analysis of the 1H NMR and HSQC spectra revealed the presence of two doublet methylene protons (δ_H_ = 3.50 y 3.22 ppm), which correlated in the HMBC spectrum to the signals of the lactone carbonyl C-1 and the oxygenated enol carbon C-3. Additionally, signals corresponding to H-23 and C-23 of anthracimycin were not observed in the spectra of **1**. Similar NMR chemical shifts reported for **3** [9], having the same substructure as **1** around the C-1 to C-3 region, confirmed our structural proposal. These evidences, together with correlations observed in the COSY and HMBC spectra of **1** (Figure 4), confirmed the planar structure of anthracimycin B as depicted in Figure 3. The similarity in magnitude and negative value of the specific rotations of **1** and **2** (${\left[\alpha \right]}_{D}^{20}$ −338.9 (**1**), −333.0 (**2**) [2]), revealed that the same absolute configuration could be proposed for these two molecules. The absolute configuration of anthracimycin had previously been determined by X-ray diffraction [3]. It is also worth mentioning that during the structural elucidation of anthracimycin B, a mistake was detected in the NMR data of anthracimycin published by Jang et al. [3], where protons attached to C-15 and C-16 seemed to be exchanged based on COSY correlations analysis. The proton correlating to the signal of the methyl group H-24 in the COSY spectrum has necessarily to be H-16, so the signal at 2.60 ppm belongs to H-16, and, therefore, the signal at 1.93 ppm to proton H-15.

Compounds **1** and **2** were tested against a panel of pathogenic bacteria consisting of four Gram-positive (methicillin sensitive and methicillin resistant *S. aureus* (MSSA and MRSA), vancomycin sensitive *Enterococcus faecium*, and vancomycin sensitive *Enterococcus faecalis*), and two Gram-negative (*Escherichia coli* and *Klebsiella pneumoniae*). Additionally, the anti-tubercular activity against *Mycobacterium tuberculosis* was also evaluated. Anthracimycin confirmed the potent activity against Gram-positive bacteria previously reported [2], with MICs in all cases below the lowest concentration tested of 0.03 μg/mL. Although less potent, anthracimycin B also displayed potent antibacterial activities with minimum inhibitory concentrations (MICs) in the submicromolar range against most of the pathogens tested (Table 2). Similar levels of activity were recently reported for compound **3** [9], perhaps indicating that, although the target and mode of action of the compound are still unknown, the presence of the methyl group at C-2 in anthracimycin is essential for its potent antimicrobial activity. Unfortunately, due to the safety level of our facilities, *B. anthracis* was not among the bacterial strains tested, but based on the previously reported activity for **2** against that pathogen, perhaps a significant level of activity for compound **1** can also be expected. Interestingly, anthracimycin also displayed activity in our study against *M. tuberculosis*, expanding the panel of antimicrobial properties of this structurally unique drug.

## 3. Discussion

Anthracimycin B, a potent antibiotic against Gram-positive pathogens, was obtained from a marine-derived actinomycete isolated from a cold-water coral collected in the Avilés Canyon (Cantabrian Sea, Northeast Atlantic Ocean). This unique coral reef ecosystem is becoming a hot spot for the discovery of novel natural products with antibiotic and cytotoxic activities. Anthracimycin B can be considered the biosynthetic precursor of anthracimycin. Its isolation from cultures of *S. cyaneofuscatus* M-169 would point out to the existence in the biosynthetic pathway of anthracimycin of a post-PKS tailoring reaction catalyzed by a free-standing S-adenosylmethionine (SAM)-dependent methyltransferase similar to CtoF in the biosynthesis of chlorotonils A and B [6]. This finding would be somehow in contradiction with the current biosynthetic proposal for anthracimycin, where the final methylation step at C-2 seems to be encoded by a methyltransferase incorporated to module 10 of the *atcF* biosynthetic gene [6,7]. Alternatively, the presence of **2** in our extracts might be the result of a malfunctioning of the methyltransferase in module 10 of the *atcF* gene, leading to the biosynthesis of a demethylated version of the drug in low quantities. Furthermore, our findings are another example of the significance of the genus, *Streptomyces*, particularly bioactive *S. cyaneofuscatus* strains from oceanic environments, for the discovery of novel natural products of medical interest. The relevance of the strain M-169 of *S. cyaneofuscatus*, in addition to the production of a new member of the anthramycin structural class with implications in the current biosynthetic pathway proposals, resides in its ability to produce high quantities (17.7 mg/L in our study) of the final compound, anthracimycin, highlighting this strain as an ideal candidate for further biosynthetic studies of this interesting antibacterial drug. Additionally, novel biological activities against *M. tuberculosis* were also detected for anthracimycin, expanding the antimicrobial potential of the molecule. Finally, although very preliminary, our study also suggests that the methyl group at C-2 present in anthracimycin plays a key role in its potent antibacterial activity.

## 4. Materials and Methods 

### 4.1. General Experimental Procedures

Optical rotations were measured using a Jasco P-2000 polarimeter (JASCO Corporation, Tokyo, Japan). UV spectra were obtained with an Agilent 1100 DAD (Agilent Technologies, Santa Clara, CA, USA). IR spectra were recorded on a JASCO FT/IR-4100 spectrometer (JASCO Corporation, Tokyo, Japan) equipped with a PIKE MIRacle^TM^ single reflection ATR accessory (PIKE Technologies Inc., Madison, WI, USA). NMR spectra were recorded on a Bruker Avance III spectrometer (500 and 125 MHz for ^1^H and ^13^C NMR, respectively) equipped with a 1.7 mm TCI MicroCryoProbe^TM^ (Bruker Biospin, Falländen, Switzerland). Chemical shifts were reported in ppm using the signals of the residual solvent as internal reference (δ_H_ 7.26 and δ_C_ 77.0 for CDCl_3_). LC–MS and LC–HRMS analyses were performed as described previously [16].

### 4.2. Taxonomic Identification of the Producing Microorganism

The strain, *S. cyaneofuscatus* M-169, was subjected to phylogenetic analysis based on 16S rDNA sequence analysis [10]. Phylogenetic analysis was performed using MEGA version 6.0 [17] after multiple alignment of data by Clustal Omega [18]. Distances (distance options according to the Kimura two-parameter model [19]) and clustering with the neighbour-joining [20] and maximum likelihood [21] methods were evaluated using bootstrap values based on 1000 replications [22]. 

### 4.3. Fermentation of the Producing Microorganism

35 petri dishes with R5A solid medium [23] (20 mL each) were inoculated with spores of strain M-169. After 6 days at 28 °C, plates were extracted with ethyl acetate. The extract was evaporated to dryness, resuspended in tert-butanol: water (1:1), and freeze dried.

### 4.4. Extraction and Isolation

The ethyl acetate extract obtained as described above was subjected to reversed phase column chromatography (Redisep Rf Reversed Phase C-18 (Teledyne Isco, Lincoln, NE, USA), 43 g, 0.040–0.063 mm). The column was eluted with H_2_O: MeOH using isocratic elution 75:25 for 10 min followed by a gradient to 100% MeOH in 35 min, a flow of 18 mL/min and UV detection at 236 and 288 nm. Fractions 13–17 containing the compounds of interest, identified by LC-MS, were pooled and subjected to semipreparative HPLC (Waters™ XBridge^®^ C_18_ (Waters Corporation, Milford, MA, USA), 10 × 150 mm, 5 µm), using a gradient H_2_O: CH_3_CN from 75 to 100% CH_3_CN in 45 min, a flow of 3.6 mL/min, and UV detection at 210 and 236 nm. Pure compounds **1** (1.0 mg, t_R_ 17.0 min) and **2** (12.4 mg, t_R_ 20.4 min) were obtained under these conditions. 

**Anthracimycin B** (**1**): White amorphous solid; ${\left[\alpha \right]}_{D}^{20}$ −338.9 (c 0.053, CHCl_3_); UV (DAD) λ_max_ 236, 288 nm; IR (ATR) ν_max_ 3014, 2959, 2925, 2873, 2855, 2832, 1740, 1715, 1606, 1456, 1376, 1280, 1250, 1215 cm^−1^; for ^1^H and ^13^C NMR data see Table 1; (+)-ESI-TOFMS *m/z* 383.220 [M + H]^+^ (calcd. for C_24_H_31_O_4_^+^, 383.2217), 400.2487 [M + NH_4_]^+^ (calcd. for C_24_H_34_NO_4_^+^, 400.2482).

### 4.5. Antibacterial Activity Assays

The antibacterial activities of the compounds were evaluated using sequential 2-fold serial dilutions of each compound in DMSO to provide 10 concentrations starting at 16 µg/mL for all the assays. Activity was measured against the strains included in Table 2 as previously reported [24,25].

## Figures and Tables

**Table 1:** NMR spectroscopic data (CDCl_3_, 500 MHz) for compound **1**.

Position	1			
	δ_C_, Type	δ_H_ (*J* in Hz)	HMBC	COSY
1	165.5, C			
2	46.5, CH_2_	3.50, d (11.3)	1, 3	2b
		3.22, d (11.3)	1, 3	2a
3	184.7, C			
3-OH		15.35, br s		
4	102.8, C	5.96, br s		
5	196.5, C			
6	53.1, CH	2.63, m	12, 14, 15, 16	7
7	37.3, CH	1.99, m	12	6, 12
8	31.2, CH_2_	2.42, br d (15.4)		8b, 9
		1.51, m	23	8a
9	120.9, CH	5.37, m	7, 11, 12, 23	8a
10	133.8, C			
11	37.3, CH_2_	2.05, m	10, 12	11b
		1.82, br d (16.9)	10, 23	11a
12	32.7, CH	2.64, m		7, 13
13	133.0, C	5.73, d (10.0)	7, 11, 12, 15, 16	12, 14
14	124.6, C	5.54, m	6, 11, 16	13
15	32.9, CH	1.96, m		6, 16
16	45.5, CH	2.65, m	14, 17, 24	15, 17, 24
17	139.0, CH	5.40, m	15, 19, 24	16, 18
18	125.9, CH	5.88, dd (10.8, 10.8)	15, 19, 20	17, 19
19	123.6, CH	6.48, dd (13.0, 12.7)	21	18, 20
20	131.3, CH	5.55, m	18, 21	19
21	70.0, CH	5.57, m		22
22	20.7, CH_3_	1.35, d (6.7)	20, 21	21
23	23.4, CH_3_	1.68, s	9, 10, 11	
24	16.2, CH_3_	0.95, d (6.0)	15, 16, 17	16

**Table 2:** MIC values of compounds **1** and **2** against bacterial pathogens.

Pathogen	Strain	MIC (µg/mL (µM))	
		Anthracimycin (1)	Anthracimycin B (2)
*S. aureus* MRSA	MB5393	<0.03 (<0.076)	0.125–0.25 (0.33–0.65)
*S. aureus* MSSA	ATCC29213	<0.03 (<0.076)	4–8 (10.5–20.9)
*E. faecium* VANS	CL144754	<0.03 (<0.076)	0.125–0.25 (0.33–0.65)
*E. faecalis* VANS	CL144492	<0.03 (<0.076)	0.25–0.5 (0.65–1.26)
*E. coli*	ATCC25922	>16 (>40.3)	>16 (>41.8)
*K. pneumoniae*	ATCC700603	>16 (>40.3)	>16 (>41.8)
*M. tuberculosis*	H37Ra	1–2 (2.5–5)	>16 (>41.8)

## Supplementary Materials

The following are available online at http://www.mdpi.com/1660-3397/16/11/406/s1, Figure S1: HPLC-UV trace, UV and ESI-TOF spectra of compound **1**; Figure S2: ^1^H NMR spectrum (CDCl_3_, 500 MHz) of compound **1**; Figure S3: ^1^H NMR (CDCl_3_, 500 MHz) of compound **1** (expansion from 13 to 17 ppm). Figure S4: ^13^C NMR spectrum (CDCl_3_, 125 MHz) of compound **1**; Figure S5: COSY spectrum of compound **1**; Figure S6: HSQC spectrum of compound **1**; Figure S7: HMBC spectrum of compound **1**; Figure S8: HPLC-UV trace, UV and ESI-TOF spectra of compound **2**; Figure S9: ^1^H NMR spectrum (CDCl_3_, 500 MHz) of compound **2**; Figure S10: ^1^H NMR (CDCl3, 500 MHz) of compound **1** (expansion from 13 to 17 ppm).
