# Supplementary material for: Anthracimycin B, a Potent Antibiotic against Gram-Positive Bacteria Isolated from Cultures of the Deep-Sea Actinomycete Streptomyces cyaneofuscatus M-169
Source: Mar Drugs. 2018 Oct 25;16(11):406. doi: 10.3390/md16110406 (PMC6267485; doi:10.3390/md16110406)
Supplement: Supplementary file 1 [file marinedrugs-16-00406-s001.pdf]

Anthracimycin B, a Potent Antibiotic against Gram-positive Bacteria isolated from Cultures of the Deep-Sea Actinomycete *Streptomyces cyaneofuscatus* M-169

Víctor Rodríguez [1], Jesús Martín [1], Aida Sarmiento-Vizcaíno [2], Mercedes de la Cruz [1], Luis A. García [3], Gloria Blanco [2,*] and Fernando Reyes [1,*]

List of supplementary materials

Figure S1. HPLC-UV trace, UV, and ESI-TOF spectra of compound **1**.

Figure S2. ^{1}H NMR spectrum ($CDCl_3$, 500 MHz) of compound **1**.

Figure S3. ^{1}H NMR ($CDCl_3$, 500 MHz) of compound **1** (expansion from 13 to 17 ppm).

Figure S4. ^{13}C NMR spectrum (DMSO-d_6, 125 MHz) of compound **1**.

Figure S5. COSY spectrum of compound **1**.

Figure S6. HSQC spectrum of compound **1**.

Figure S7. HMBC spectrum of compound **1**.

Figure S8. HPLC-UV trace, UV, and ESI-TOF spectra of compound **1**.

Figure S9. ^{1}H NMR spectrum ($CDCl_3$, 500 MHz) of compound **2**.

Figure S10. ^{1}H NMR ($CDCl_3$, 500 MHz) of compound **1** (expansion from 13 to 17 ppm).

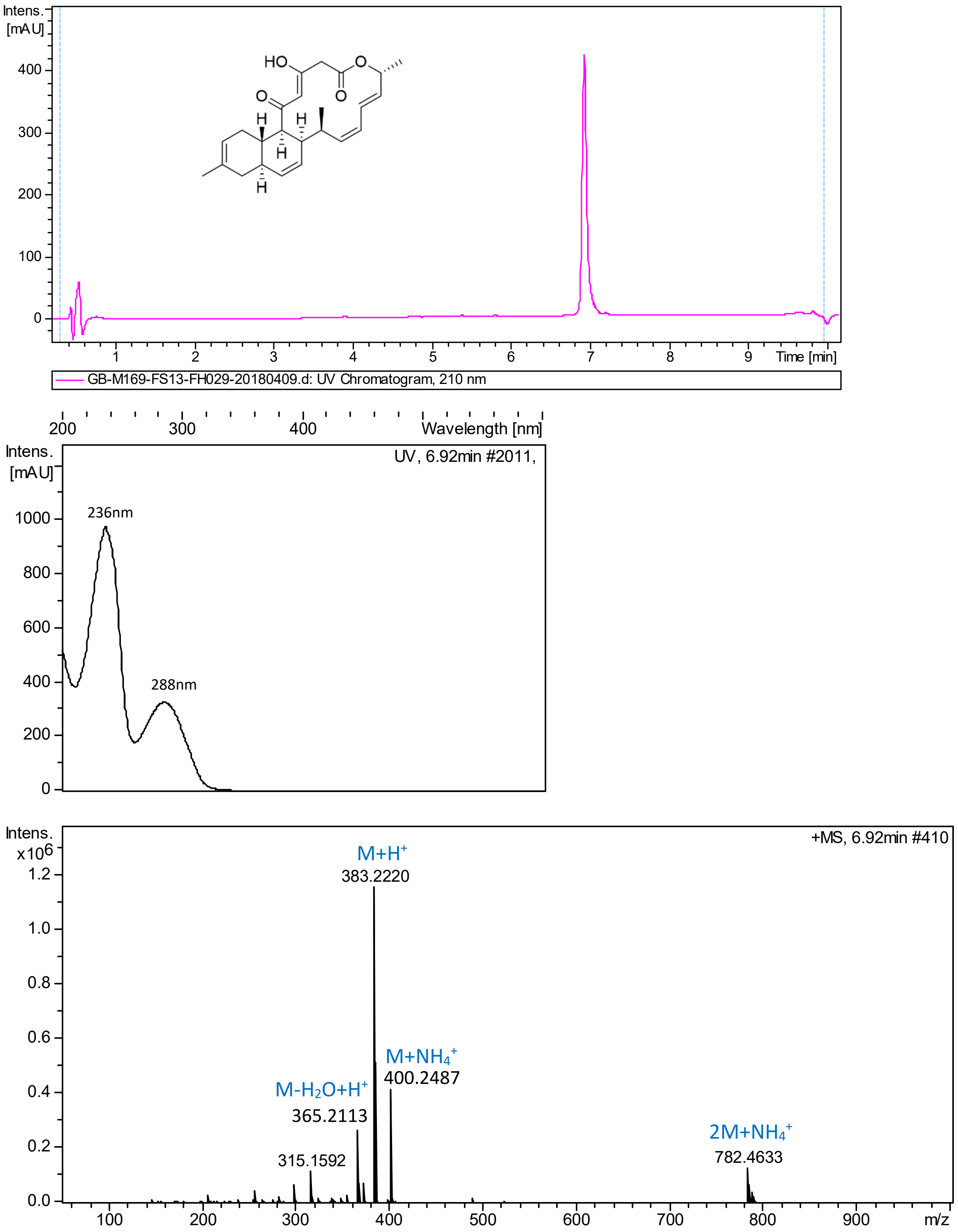

Figure S1. HPLC-UV trace, UV, and ESI-TOF spectrum of compound **1**.

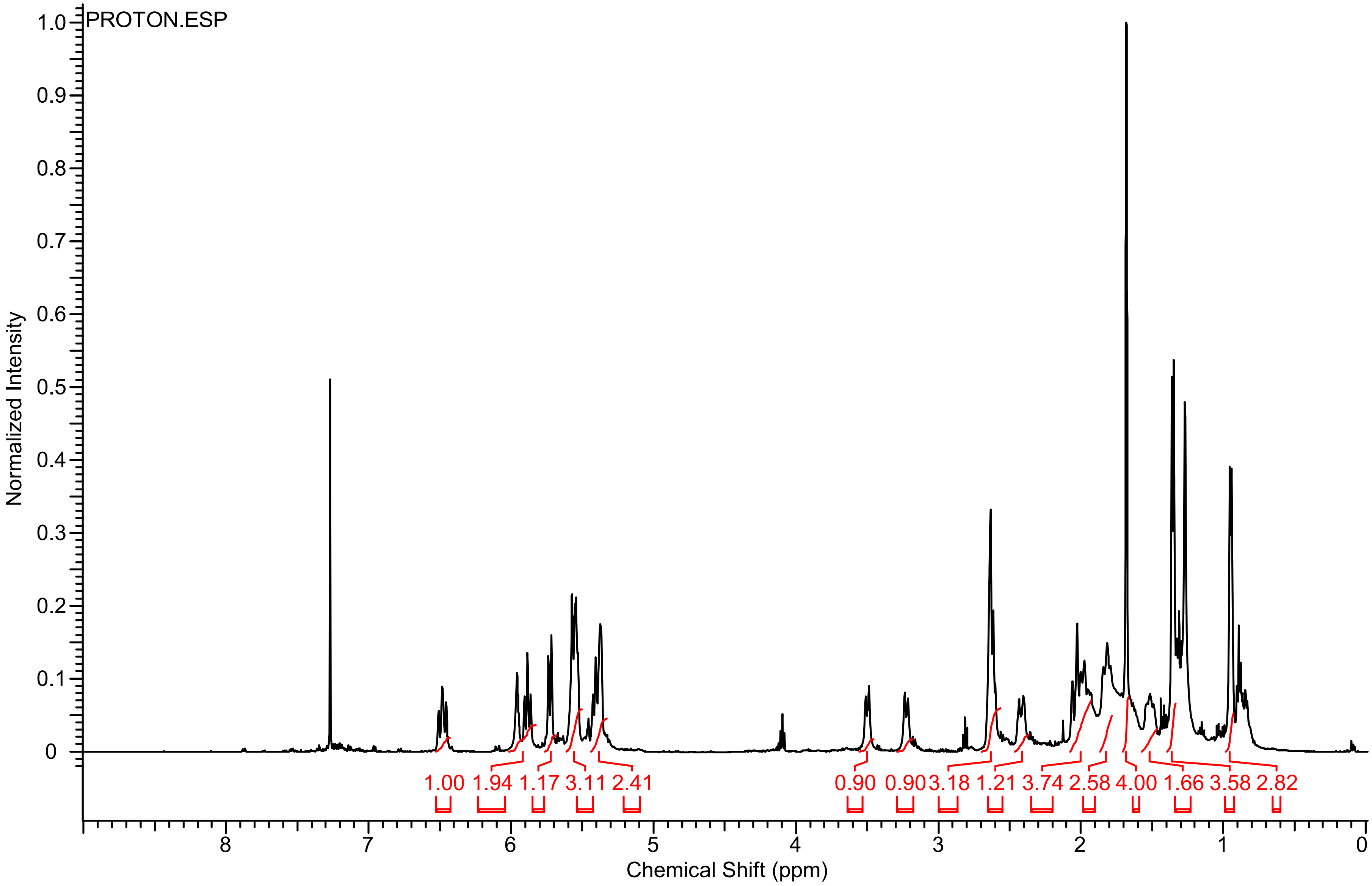

Figure S2. ^{1}H NMR ($CDCl_3$, 500 MHz) of compound **1**.

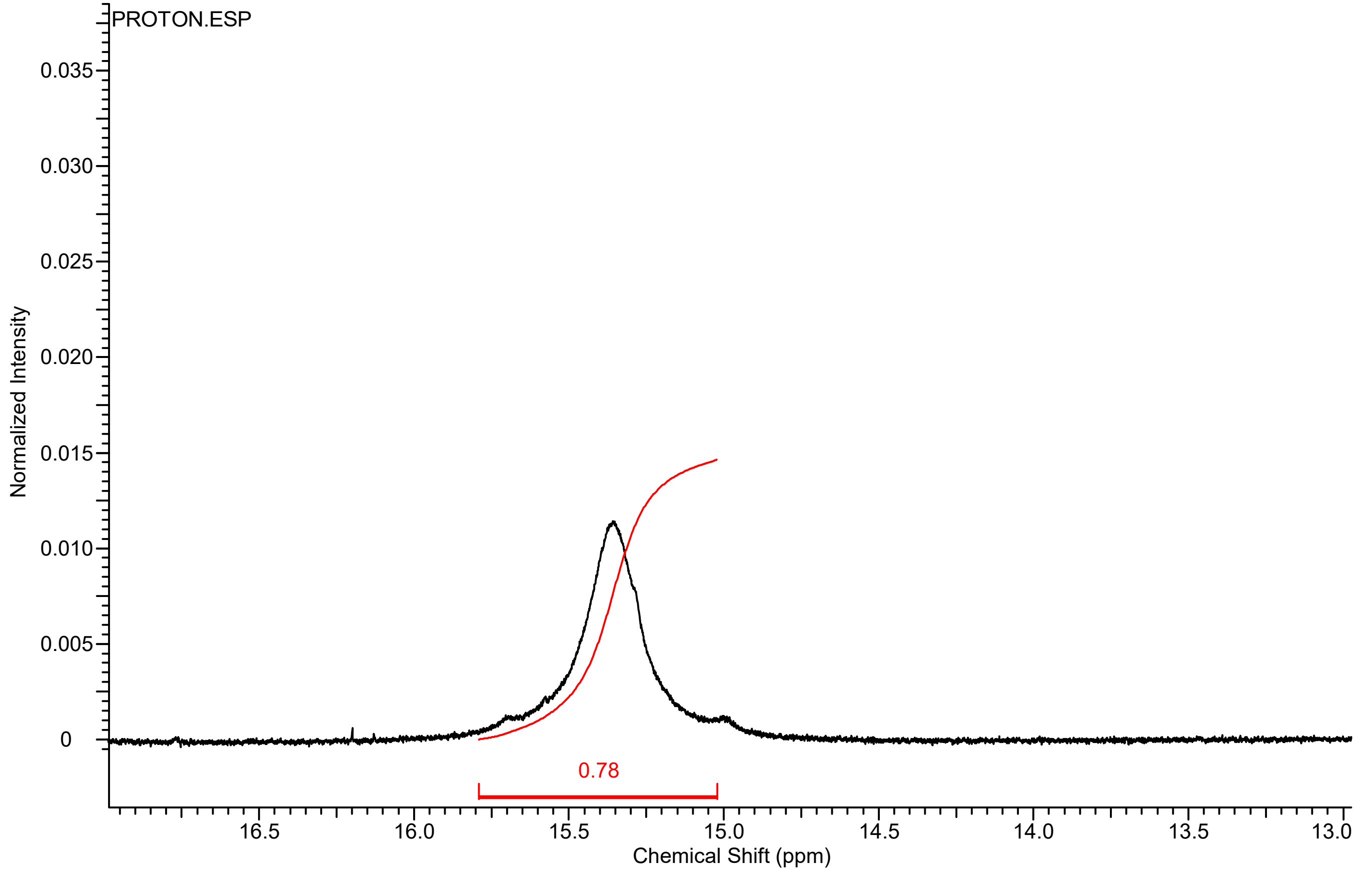

Figure S3. ^{1}H NMR ($CDCl_3$, 500 MHz) of compound **1** (expansion from 13 to 17 ppm).

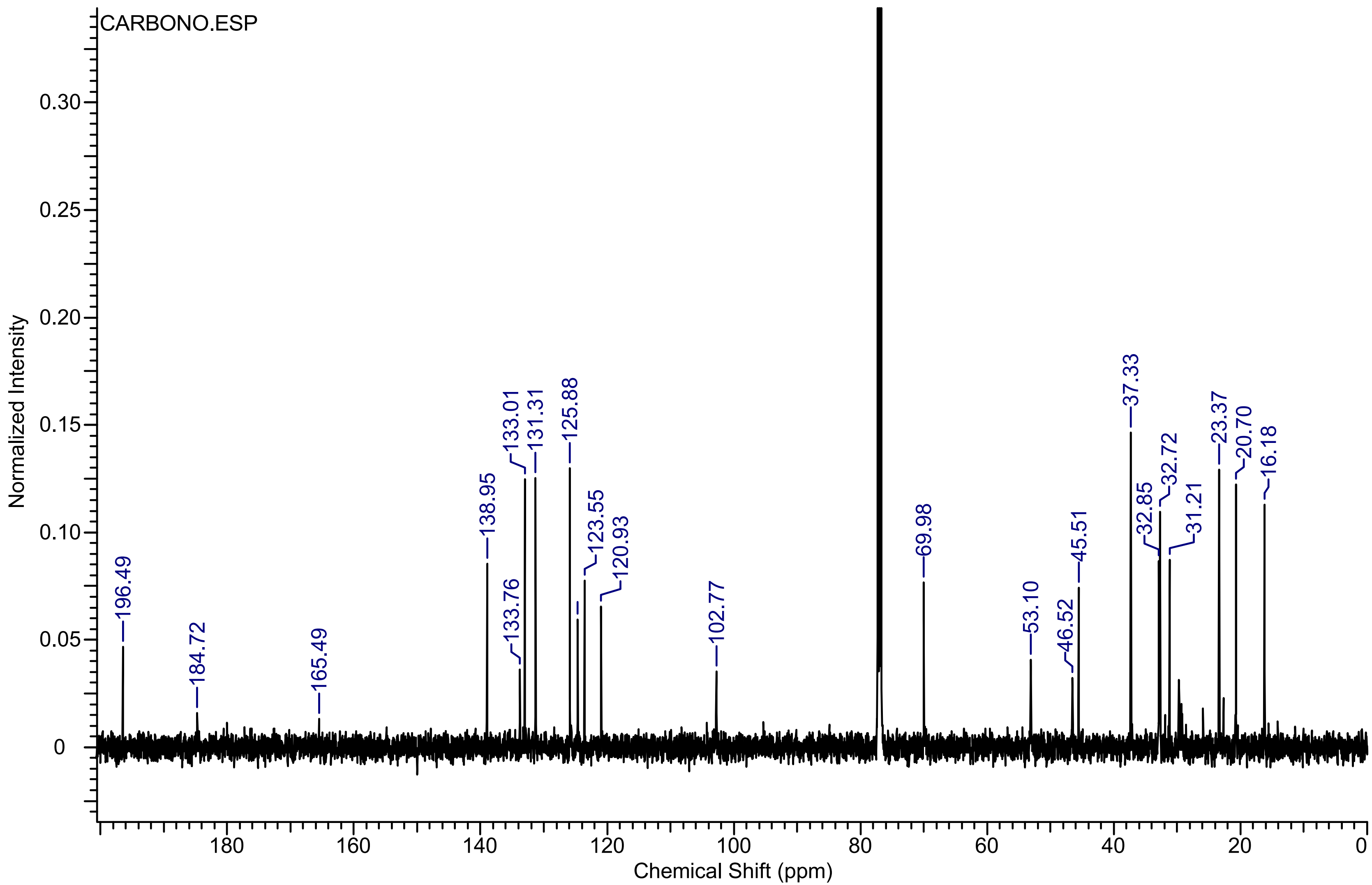

Figure S4. ^{13}C NMR ($CDCl_3$, 125 MHz) of compound **1**.

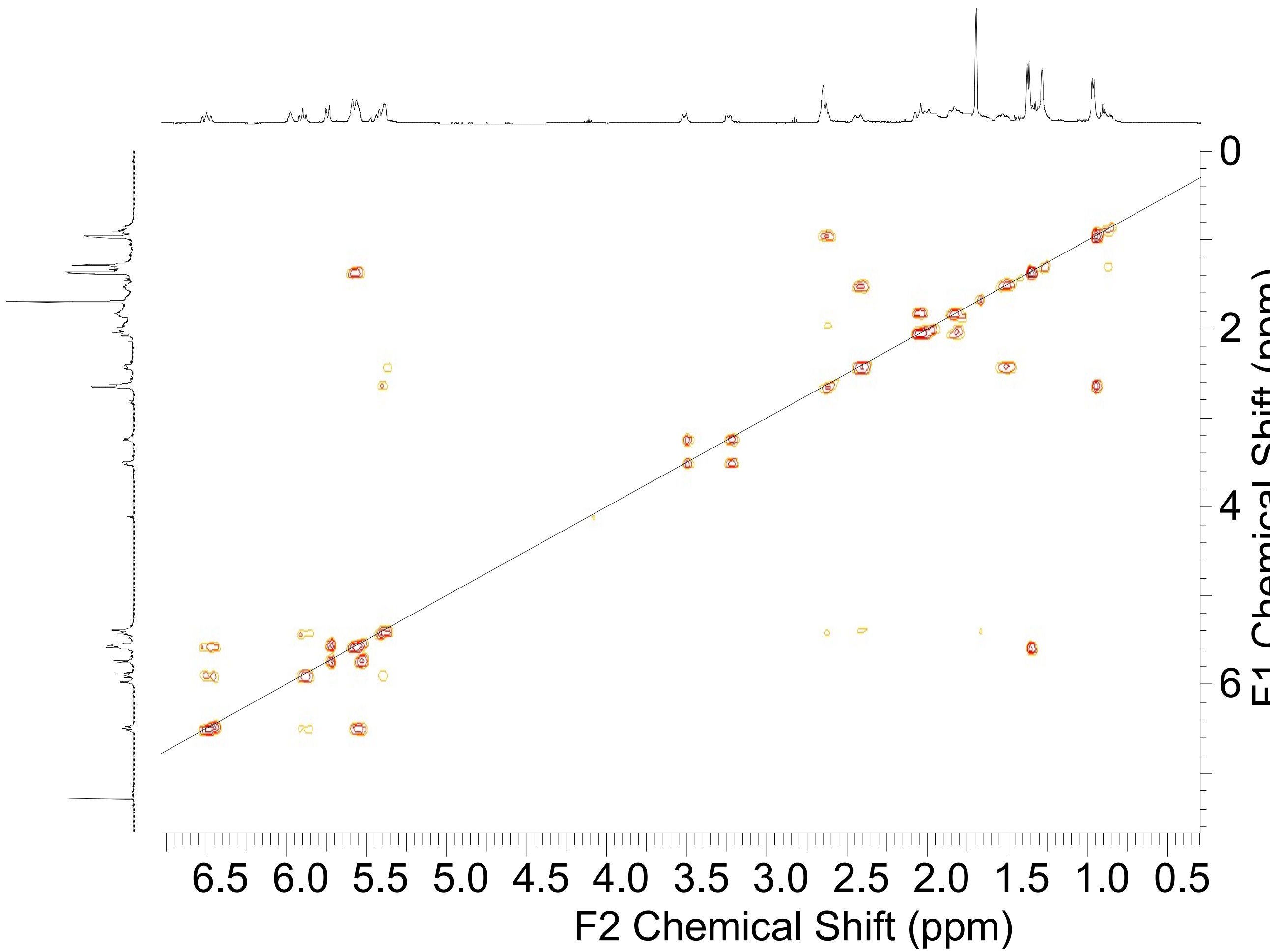

Figure S5. COSY spectrum of compound **1**.

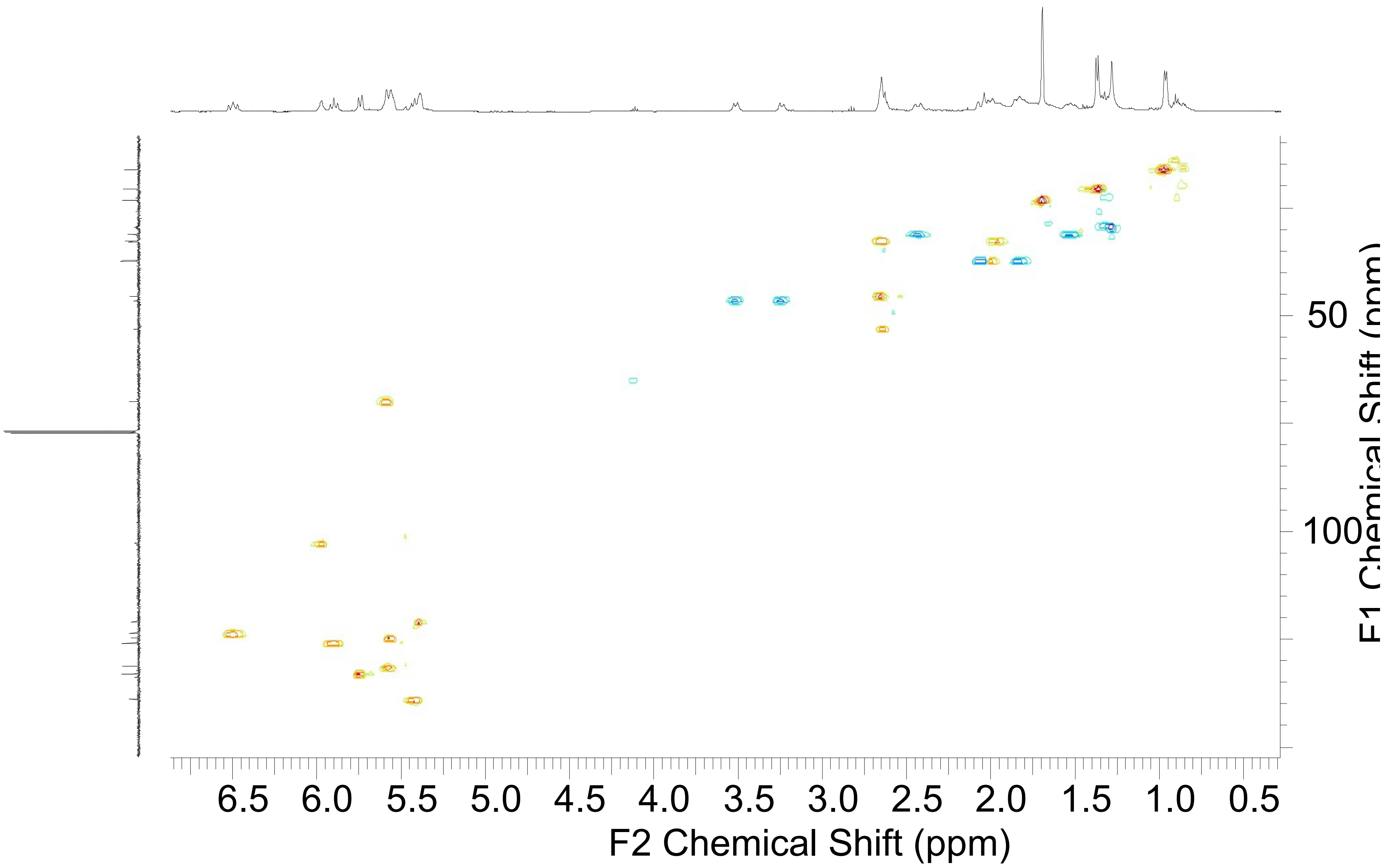

Figure S6. HSQC spectrum of compound **1**.

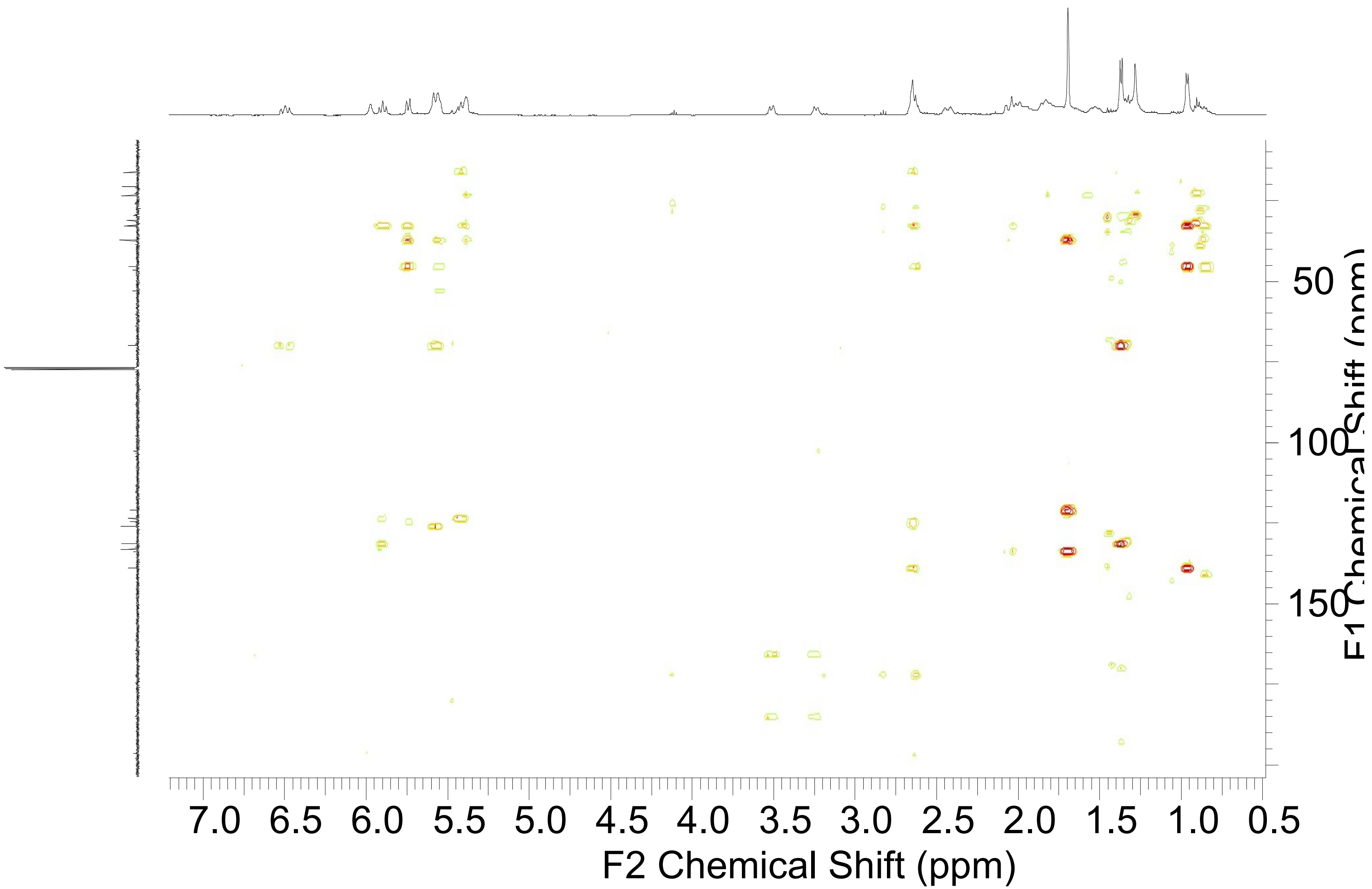

Figure S7. HMBC spectrum of compound **1**.

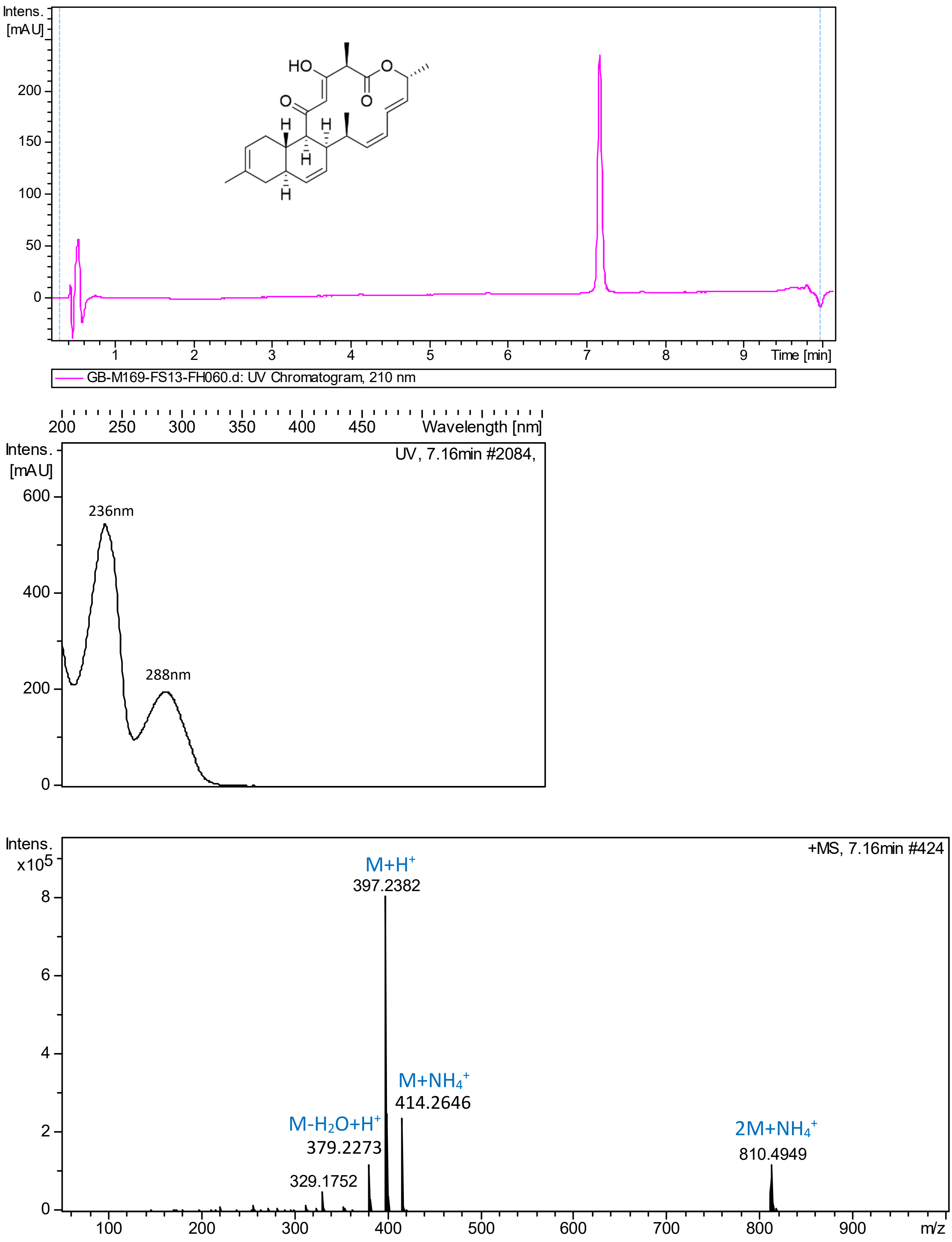

Figure S8. HPLC-UV trace, UV, and ESI-TOF spectrum of compound **2**.

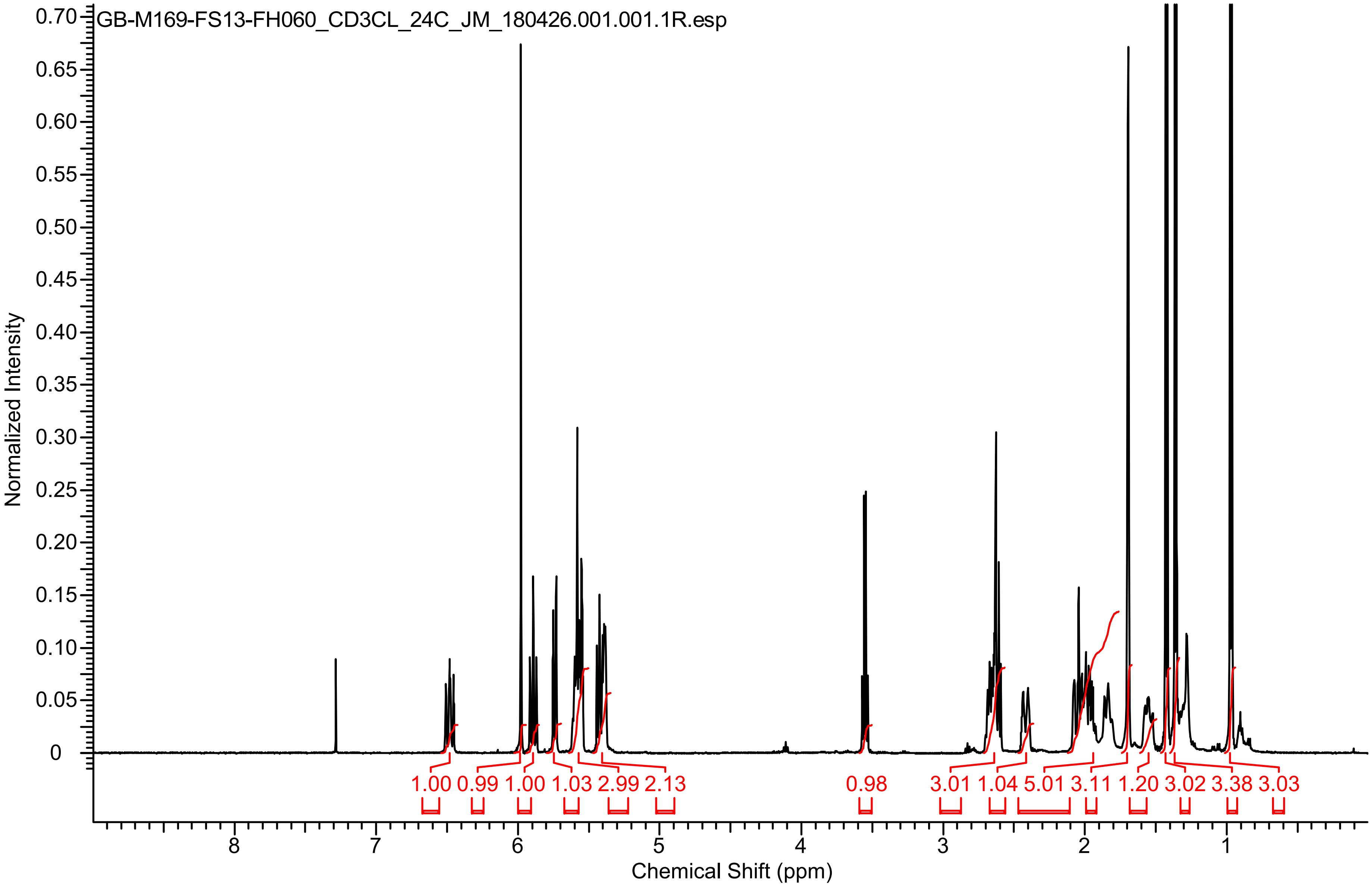

Figure S9. ^{1}H NMR ($CDCl_3$, 500 MHz) of compound **2**.

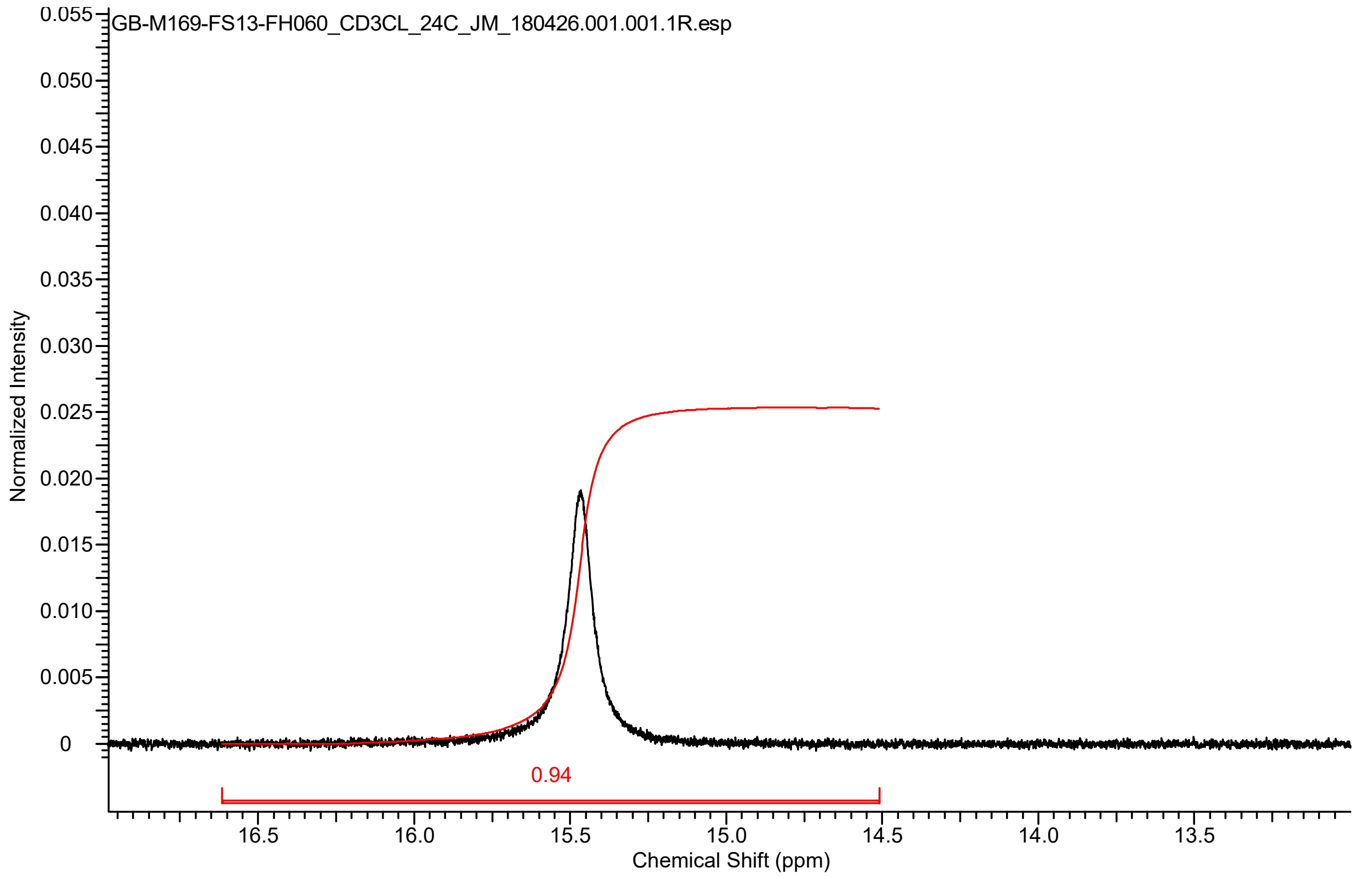

Figure S10. ^{1}H NMR ($CDCl_3$, 500 MHz) of compound **2** (expansion from 13 to 17 ppm).
